# Large-Scale Cloning and Comparative Analysis of TaNAC Genes in Response to Stripe Rust and Powdery Mildew in Wheat (*Triticum aestivum* L.)

**DOI:** 10.3390/genes11091073

**Published:** 2020-09-12

**Authors:** Shikai Lv, Huan Guo, Min Zhang, Qiaohui Wang, Hong Zhang, Wanquan Ji

**Affiliations:** 1 State Key Laboratory of Crop Stress Biology for Arid Areas, College of Agronomy, Northwest A&F University, Yangling 712100, China; lvshikaiyd@nwafu.edu.cn (S.L.); guohuan2018@163.com (H.G.); Zhangm9977@163.com (M.Z.); nxwywjy@163.com (Q.W.); 2 Shaanxi Research Station of Crop Gene Resources and Germplasm Enhancement, Ministry of Agriculture, Yangling 712100, China

**Keywords:** TaNAC, wheat, stripe rust, powdery mildew, cloning, comparative analysis, alternative splicing, structural variants

## Abstract

The NAM, ATAF1/2, and CUC2 (NAC) transcription factors (TFs) constitute the largest plant-specific TF superfamily, and play important roles in various physiological processes, including stress responses. Stripe rust and powdery mildew are the most damaging of the fungal diseases that afflict wheat (*Triticum aestivum* L.). However, studies on *Triticum aestivum NAC* (*TaNAC*)*s*’ role in resistance to the two diseases are still limited, especially in an overall comparative analysis of *TaNACs* responding or not to fungal stress. In the present study, 186 TaNAC transcripts were obtained from the resistant hexaploid wheat line N9134 under fungal stress, and 180 new transcripts were submitted to GenBank. Statistical results show that 35.1% (54/154) of TaNAC genes responded to stripe rust and powdery mildew in the seedling stage. “Abnormal” coding transcripts of differentially expressed (DE)-TaNAC genes in wheat responding to fungal stress were found in a significantly higher proportion (24/117 vs. 8/69, *p* = 0.0098) than in non-DE-NACs. This hinted that the alternative splicing of TaNAC genes was active in transcriptional or post-transcriptional regulation during plant-pathogen interactions. Full-length NAC proteins were classified into nine groups via phylogenetic analysis. Multiple-sequence alignment revealed diversity in the C-terminal structural organization, but the differentially expressed gene (DEG)-encoding proteins enriched in Subgroups VI and VII were conserved, with WV[L/V]CR amino acid residues in Motif 7 following the NAM domain. Our data that showed TaNAC TFs responded to fungal disease, which was affected by expression levels and by the regulation of multifarious transcript variants. These data for TaNAC responses to stripe rust and/or powdery mildew and their numerous structural variants provide a good resource for NAC function–mechanism analysis in the context of biotic-stress tolerance in wheat.

## 1. Introduction

The NAC (NAM, ATAF1/2, CUC2) transcription-factor (TF) family is one of the largest plant-specific TF families [1], containing consensus sequences from *No Apical Meristem (NAM*) in petunias (*Petunia hybrida*), and *ATAF1*,*2* and *Cup-Shaped Cotyledon (CUC2*) in *Arabidopsis* (*Arabidopsis thaliana*) [2,3]. NAC proteins are characterized by a highly conserved DNA-binding domain at the N terminus, while a flexible C-terminal domain is usually essential for transcriptional activation [4,5]. TFs cooperate with corresponding cis-regulatory sequences and then act as molecular switches to regulate downstream gene expression [6]. Numerous studies have evidenced that NAC TFs have functions in nearly all organizations and tissue types, and are implicated in every physiological process in the plant life cycle, including morphogenesis, development, senescence, and plant adaptation to environmental stress [7,8,9,10,11]. For example, in *Arabidopsis*, NAC25 and NAC1L were identified as upstream regulators of cell-expansion gene expression, gibberellin-mediated endosperm expansion, and seed germination [12], while ANAC096 cooperates with bZIPs to alleviate dehydration and osmotic stress [13]. In rice, OsNAP connects abscisic acid and leaf senescence by fine-tuning abscisic-acid biosynthesis and directly targeting senescence-associated genes [14]. As transcription factors, NAC TFs play a fundamental role in regulating gene expression in plant growth and development and in response to environmental stimuli [9,10]. As genes, promoters of NAC genes similarly contain binding sites of TFs, such as W-boxes, GCC-boxes, and MYB, which bind to WRKY, ERF, and MYB TFs, respectively [15,16,17]. This demonstrates that transcription factors have the capacity to regulate the expression of each other by manipulating a net of gene expressions. The investigation of NAC family is therefore an important area of postgenomic research on abiotic and biotic stress. 

Wheat is one of the most important crops globally, with a large complex genome. The number of NAC TF-encoding genes in wheat is much larger than in the model plant *Arabidopsis* (138) or diploid rice (*Oryza sativa,* 170), [18,19]. Recently, the NAC TF family of hexaploid wheat was categorized on the basis of the wheat reference genome TGAC and the IWGS RefSeq v1.0 database [20,21]. A total of 488 full-length sequences containing the NAC domain were annotated (460 high- and 28 low-confidence sequences) [20]. Stripe rust and powdery mildew, caused by *Puccinia striiformis Westend.* f. sp. *Tritici Eriks.* (*Pst*) and *Blumeria graminis* f. sp. *tritici* (*Bgt*), respectively, are two of the most damaging wheat diseases globally [22]. Several reports have indicated that NAC TFs play crucial roles in the transcriptional reprogramming associated with plants’ innate immune system, basal defense, and systemic acquired resistance [23,24]. The *HvNAC6* gene was proven to be a positive regulator in the basal defense of penetration resistance when barley was inoculated with virulent isolates of *Blumeria graminis f.sp. hordei* [25]. In rice, overexpression of *OsNAC4* led to hypersensitive-response (HR) cell death; in *OsNAC4* knocked-down lines, HR cell death was markedly decreased in response to an avirulent bacterial strain [26]. These results indicate that NAC TFs are involved in responding to fungal diseases, and provide direction for further investigation of the mechanism by which NAC regulates resistance-gene expression in wheat. Unfortunately, to date, only a limited number of NAC TFs that function against fungi in wheat have been reported in detail [27,28,29].

In the present study, large-scale cloning of *Triticum aestivum* NAC (TaNAC) genes was carried out on the basis of RT-PCR and RNA-seq data in wheat undergoing *Pst* and/or *Bgt* stress, while the expression of full-length transcripts was verified using qRT-PCR. Subsequently, 186 TaNACs were clustered and systematically analyzed for phylogenetic classification, conserved domains/motifs, and transmembrane motifs. To better understand gene function, we further investigated the structural characteristics of genes that significantly contrasted with that displayed nonsignificant differential expression (DE) under the fungal treatments. This provides a theoretical reference for wheat resistance-regulating-gene mining and stress-resistance breeding.

## 2. Materials and Methods

### 2.1. Plant Materials and Fungi

Winter wheat line N9134, developed at Northwest A&F University, shows high resistance to *Pst* race CYR 31 and is also resistant to all *Bgt* races due to two individual genetic resistance loci on Chromosomes 1B and 5BL, respectively [30]. *Pst* race CYR 31 and *Bgt* isolate E09 were maintained by the College of Plant Protection and the College of Agronomy of Northwest A&F University, respectively. N9134 plants were cultivated in soil in a growth chamber at 18 °C under a 16 hours light/8 hours dark photoperiod. Fungal inoculation was performed as previously described [30]. At about 7 days old, seedlings were divided into two groups and inoculated with either *Bgt* E09 or *Pst* race CYR 31 conidia. Freshly collected spores were inoculated onto the surfaces of the primary leaves with a paintbrush. Highly susceptible common wheat varieties, Shaanyou 225 and Huixianhong, were inoculated with E09 and CYR 31 in the same way to check the inoculation effect. The test was conducted with three biological replications. The infected leaves of N9134 were harvested at 0, 12, 24, 36, 48, 72, 96, and 120 h after being inoculated, frozen immediately in liquid nitrogen, and stored at −80 °C.

### 2.2. Reidentification and Sequence Analyses of NAC TFs in Wheat

To obtain full-scale information regarding wheat NAC TFs, we employed and integrated the wheat reference genome database, NAC TF database of *A. thaliana* (mouse-ear cress), and *Oryza sativa* subsp. *Japonica*. IWGSC RefSeq v1.1 (133,744 HC sequences) was downloaded from URGI (https://wheat-urgi.versailles.inra.fr/) [31]. The NAC TFs of *A. thaliana* and *Oryza sativa* were downloaded from PlantTFDB v5.0 (http://planttfdb.cbi.pku.edu.cn/index.php) and UniProtKB (https://www.uniprot.org/uniprot/). After deredundancy, 133 AtNAC and 172 OsNAC TF unique sequences were used to search TaNAC TFs by local BLAST alignment with the peptide sequences in the IWGSC RefSeq v1.1. Furthermore, BLASTP was used to filter out low-confidence sequences in the NCBI website (https://www.ncbi.nlm.nih.gov/) by selecting the Swissport database. Additionally, all identified NAC candidate genes were searched for conserved domains using Conserved Domain Search Service (CD Search) software (https://www.ncbi.nlm.nih.gov/Structure/cdd/wrpsb.cgi) and the SMART tool (http://smart.embl-heidelberg.de/) to confirm the reliability of sequences. Both operations were performed under default parameter settings.

### 2.3. RNA Extraction

Total RNA was extracted from the specified time-point samples using a Trizol reagent (Invitrogen, Carlsbad, CA, USA) in accordance with the manufacturer’s instructions, but with a few modifications regarding DNase digestion and RNA purification. Unless otherwise specified, each experiment procedure was carried out strictly in accordance with the manufacturer’s instructions regarding the instrument and reagents. A small fraction of RNA was electrophoresed in 1% agarose gel to check its quality. Furthermore, the concentration of RNA samples was examined by ultra-micro-spectrophotometer (Nanodrop ND-1000; Nanodrop Technologies, Wilmington, DE, USA). 

### 2.4. Screening Fungus-Responsive NAC TFs and Evaluating Splice Variants

On the basis of the RNA-seq data of N9134 [30], we screened all expressed sequences annotated as NAC TFs. The reads per kilobase of transcript per million mapped (RPKM) value extracted from the original data was directly employed to analyze and calculate the expression. All transcribed genes had to have one mRNA transcript in different conditions with a value of RPKM ≥ 1 (i.e., the transcript was considered reliable). Unigenes with log2 fold change (log 2 FC) > 1 or < −1 and FDR at 1.0% were determined as differentially expressed transcripts in response to *Pst* and *Bgt* stress versus mock inoculation. The cDNA template was synthesized from an equal proportional mixture of 16 total RNAs that were extracted from each sample of the eight time points under *Pst* and *Bgt* treatment, respectively. PCR amplification was performed using PrimeSTAR HS DNA Polymerase (Takara, Dalian, China). Primers (Supplementary Table S5) were designed with Primer 5.0 software. PCR products were cloned into the pMD19-T Vector (Takara, Dalian, China). Sequencing of the cloned fragment and primer synthesis were performed in Beijing AuGCT DNA-SYN Biotechnology Co. (Beijing, China).

### 2.5. Real-Time Quantitative PCR Analysis

Purified total RNAs were reversed to cDNA with PrimeScript RT reagent Kit (Perfect Real-Time) (Takara, Dalian, China). Quantitative RT-PCR analysis was then carried out using SYBR Premix Ex Taq II (Tli RNaseH Plus) (Takara, Dalian, China) on Applied Biosystems QuantStudio 7 (or 3) Real-Time PCR (Life Technologies, Foster, CA, USA). Wheat α-tubulin gene was used as the internal reference gene for normalization of the qRT-PCR data. PCR efficiency (95%–105%) was verified. The qRT-PCR assay was carried out using a mixed sample of three seedlings and three technical replicates. Primers are listed in Supplementary Table S6. Relative expression was calculated using the comparative cycle threshold and the 2^−ΔΔCT^ method [32]. 

### 2.6. Analysis of TaNAC TF Protein-Sequence Characteristics

The theoretical physicochemical properties of TaNAC TFs were analyzed by uploading peptide sequences to ExPASy using the ProtParam tool with default settings (https://web.expasy.org/protparam/). The transmembrane motifs and subcellular localization of TaNAC TFs were predicted using TMHMM server v.2.0 (https://services.healthtech.dtu.dk/service.php?TMHMM-2.0) and CELLO server v.2.5 (http://cello.life.nctu.edu.tw/), respectively. Multiple Expectation Maximization for Motif Elicitation (MEME) suite (version 5.1.1; http://meme-suite.org/tools/meme) was employed to identify the conserved motifs, and the parameters were the default except for the number of repetitions, set as 0 or 1 site per sequence; maximal number of motifs set as 20; and optimal width of each motif set as from 6 to 80 residues. 

For phylogenetic analysis and comparing the structural features of TaNAC TFs that responded to *Pst* and *Bgt* stresses, the deduced amino acid sequences of the two types of TaNAC transcripts, responding to fungal stress or not, were aligned using the MUSCLE program with default parameters [33]. Molecular Evolutionary Genetic Analysis (MEGA) software version X was used to conduct evolutionary analyses and generate a phylogenetic tree [34]. The evolutionary history was inferred using the maximum-likelihood method and JTT matrix-based model [35]. Discrete gamma distribution was used to model evolutionary-rate differences among sites (five categories). All positions with less than 95% site coverage were eliminated (partial-deletion option). The bootstrap consensus tree inferred from 1000 replicates was taken to represent the evolutionary history of the analyzed taxa [36].

## 3. Results

### 3.1. Genome-Wide Reidentification of TaNAC TFs in IWGSC RefSeq v1.1

Considering the updated reference-genome data, we reidentified NAC TFs in wheat. Here, a total of 101 high-confidence (HC) NAC transcripts were added, which raised the number to 559 of TaNAC transcripts from 460 gene loci (Supplementary Table S1). In comparison with the previous report by Guerin et al. (2019), two genes, namely TraesCS1D02G004800 and TraesCS5B02G271800, were supplemented, while an additional 99 transcripts were detected from 66 TaNAC genes in IWGSC RefSeq v1.1 due to different splicing. Alignment results show that 11 transcripts of nine location-unknown TaNAC genes could be classified into three homologous groups, of which four (TraesCSU02G137200, TraesCSU02G163300, TraesCSU02G168500, and TraesCSU02G215800), three (TraesCSU02G119900, TraesCSU02G120000, and TraesCSU02G174800), and two (TraesCSU02G135000 and TraesCSU02G230600) genes were located in the second, sixth, and seventh homologous groups, respectively.

As shown in Table 1, 22 TaNAC transcripts had exceptional domain structures. For example, three transcripts did not include the conserved domain, while eight transcripts had only a short or partial NAM domain. Further analysis of these 11 transcripts revealed that TraesCS5A02G271500.1, TraesCS5D02G279100.1, TraesCS2A02G326600.3, TraesCS2D02G336300.2, TraesCS5B02G480900.1, and TraesCS5D02G481200.2 were formed by alternative splicing (AS). The other corresponding transcripts of the same gene had complete NAC conserved domains, which indicated that these transcripts belonged to loss-functional splicing variants or pseudogenes. Our investigation also found that two transcripts contained other domains besides the normal NAC domain. Briefly, TraesCS1D02G004800.1 had the DnaJ and ZnF_BED domains, while TraesCS7B02G461700.1 harbored two consecutive AA_Kinase domains. Additionally, eight transcripts all had two complete NAM domains, while another had an N-terminal signal peptide composed of 16 amino acids (aa).

### 3.2. Cloning and Nomenclature of New TaNAC Transcripts in N9134

**Figure 1:**
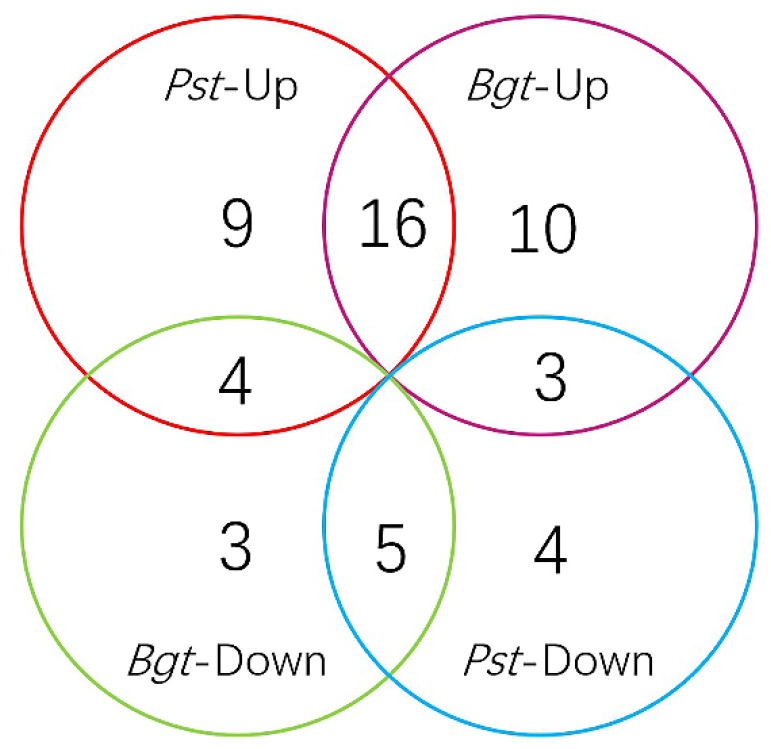
Distribution of differentially expressed (DE) *Triticum aestivum* NAC (TaNAC) unigenes under stress from stripe rust and powdery mildew.

To understand the roles of TaNAC TFs in wheat responding to pathogen stress, we focused on genome-wide TaNAC genes on the basis of previous RNA-seq data (PRJNA243835) [30]. According to the annotation of the Swiss-Prot or NR database, 154 NAC candidate unigenes were obtained after eliminating redundant sequences. Among them, 54 were identified as differentially expressed genes (DEGs) in response to *Pst* and/or *Bgt* stress (Supplementary Table S2). Twenty-nine TaNAC unigenes were upregulated and 12 were downregulated under *Pst* stress. Coincidentally, the same numbers of TaNAC genes were up- or downregulated under *Bgt* stress. Our results show that 28 unigenes were significantly differentially expressed under both *Pst* and *Bgt* stress (Figure 1).

Furthermore, we randomly selected 28 DE-NACs (unigenes) and 22 non-DEs as representatives (Supplementary Table S3) and compared then to clarify the divergence of sequence structure. Considering the variety of wheats’ genetic backgrounds, we captured the full-length transcripts and splicing variants from N9134 infected by *Pst* and *Bgt* using RT-PCR and/or reassembly. Here, a total of 186 full-length TaNAC transcripts (167 of them were cloned) were obtained, which could belong to 84 gene loci (77 of them were cloned) and included 154 normal coding sequences and 32 “abnormal” coding sequences (Supplementary Table S3), of which 180 new transcripts were submitted to the GenBank database (accession nos. KY461014, KY461022, KY461025, KY461026, KY461030, KY461031, KY461035, KY461040, KY461041, KY461043, KY461052, MN747(172–307), and MN7864(10–42).). Hereafter, for descriptive convenience, they are named according to similarity with homologous sequences in other species determined using Blastx.

The list of these 186 TaNAC transcripts is given in Supplementary Table S3, as well as their structural characterizations, predicted subcellular localizations, and theoretical physicochemical properties, including aliphatic index, grand average of hydropathicity (GRAVY), instability index, molecular weight (average), and theoretical pI. Comparing the information of these fungus-stressed TaNACs (Supplementary Table S3) with 559 high-quality TaNAC transcripts from IWGSC RefSeq v1.1 (Supplementary Table S1), we found that they had similar physiological and biochemical characteristics. 

### 3.3. TaNAC Expression in Wheat under Fungus Infection

**Figure 2:**
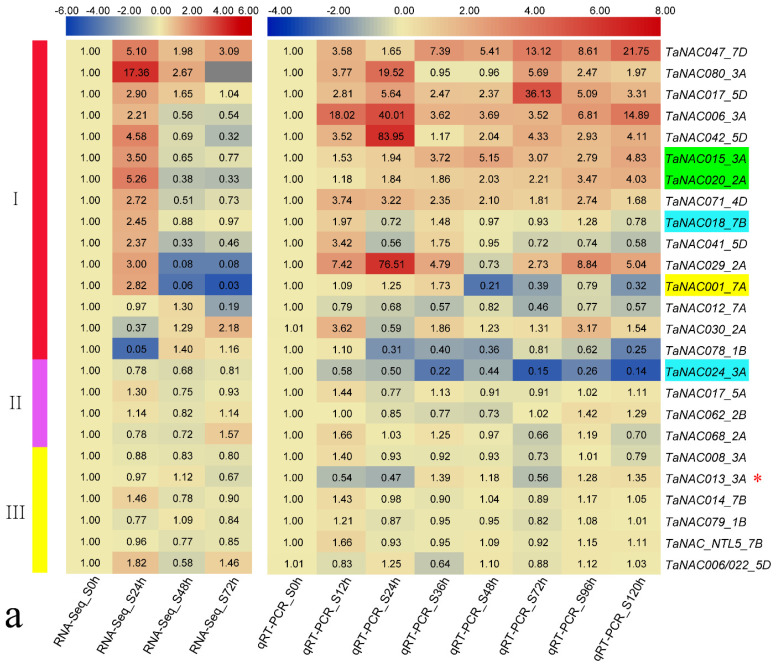

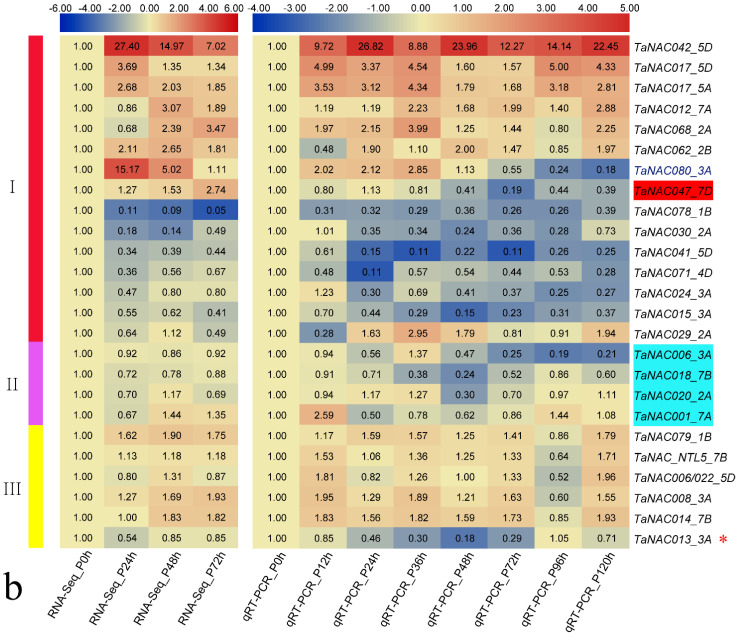
Expression heat map of 25 selected TaNAC genes under stress due to stripe rust or powdery mildew. (**a**) *Pst* treatment; (**b**) *Bgt* treatment. (left) Expression determined by RNA-seq, and values represent fold change of average reads per kilobase of transcript per million mapped (RPKM) across three replicate samples; (right) expression of corresponding TaNAC genes according to qRT-PCR, and value is relative expression calculated using 2^−ΔΔCt^. S: stripe-rust stress; P: powdery-mildew stress; 0, 24 h, etc.: time interval between sampling and inoculation. Group I contained genes that were differentially expressed under both *Pst* and *Bgt* stress. (**a**) Group II contained genes that were non-DE under *Pst* stress, but DE under *Bgt* stress; (**b**) Group II contained genes that were non-DE under *Bgt* stress, but DE under *Pst* stress; genes in Group II were determined to be DE under all fungal stress. Group III contained genes that were non-DE under both *Pst* and *Bgt* stress. Notes: TaNAC015_3A and TaNAC020_2A, marked in green in Figure 2a, were upregulated both in RNA-seq and qRT-PCR but with inconsistent expression patterns; TaNAC001_7A, marked in yellow in Figure 2a, was upregulated in RNA-seq and downregulated in qRT-PCR, but with consistent expression patterns; TaNAC047_7D, marked in red in Figure 2b, was upregulated in RNA-seq and downregulated in qRT-PCR, and with inconsistent expression patterns. The four sets of comparison result above showed no change in DE classification attribute. Six other sets of comparison results, marked in blue—namely TaNAC018_7B and TaNAC024_3A in Figure 2a, and TaNAC006_3A, TaNAC018_7B, TaNAC020_2A, and TaNAC001_7A in Figure 2b—were non-DE in RNA-seq but DE in qRT-PCR, or the opposite. They all showed inconsistent expression patterns. However, upon combining the two sets of RNA-seq and qRT-PCR comparison results between *Pst* stress and *Bgt* stress, respectively, there was no change in DE classification attribute under fungal stress for any gene. In addition, only TaNAC013_3A.1, marked with a red star, showed a changed classification attribute from non-DE in RNA-seq to DE in qRT-PCR, with inconsistency of specific expression patterns under fungal stress.

Of the 84 genes from N9134, 25 were randomly chosen as representatives for a qRT-PCR experiment to verify the reliability of the differentially expressed (DE) classification of the RNA-seq results. The results (Figure 2) show expression levels in RNA-seq and qRT-PCR at different times in N9134 under *Pst* or *Bgt* stress, which also allowed the direct comparison of the DE/non-DE classification attributes, and the specific expression patterns between the two experiment methods. A total of 38 sets (unlabeled in Figure 2) exhibited good consistency between qRT-PCR and RNA-seq analyses in both the DE/non-DE classification attributes and the specific expression patterns under two types of fungal stress, accounting for 76.0% (38 out of 50) of all comparison sets. In the 10 other sets of comparison (labeled by four colors in Figure 2), the classification attribute of DE/non-DE under fungal stress was consistent between the two experiment methods, although the expression patterns fluctuated a little, or were an imperfect match under either *Pst* or *Bgt* stress. 

For the DE/non-DE classification attribute of TaNAC genes under fungal stress, 24 of the 25 selected TaNAC genes (apart from TaNAC013_3A marked with star in Figure 2) showed good consistency between qRT-PCR results and previous RNA-seq results, which meant that the ratio of conformity reached 96.0% (24 out of 25). Therefore, this substantiated the DE/non-DE classification attributes of TaNAC genes under fungal stress using RNA-seq, which guaranteed subsequent analyses.

### 3.4. Structural Variations of TaNAC Transcripts in N9134 under Fungal Stress

Compared to annotated transcripts in the reference genome, we found that of the 186 TaNAC transcripts that were transcribed from 84 gene loci, at least 102 had structural variation. Meanwhile, of the 77 cloned genes, 30 had more transcripts than the corresponding genes in IWGSC RefSeq v1.1, and the number of extra transcripts was 79. Interestingly, of the 186 TaNAC transcripts, 154 could still be normally encoded without frameshift or premature termination, although alternative splicing (23 of them) and other structural modifications were involved. In addition, the 32 other transcripts were all “abnormal” coding sequences due to structural variations, including single-nucleotide variation (SNV), small insertions and deletions (s-InDel; length of the involved fragment was 3–30 bp), and insertion or deletion of large fragments with or without the typical intron-boundary mark. Of these 32, 25 were caused by alternative splicing. These “abnormal” coding sequences could encode segmented or partial NAC normal proteins, but with a small fragment of shifted amino acids in the N- or C-terminal. Detailed variants of the 186 transcripts obtained from the leaves of N9134 undergoing fungal stress can be found in Supplementary Table S3.

**Figure 3:**
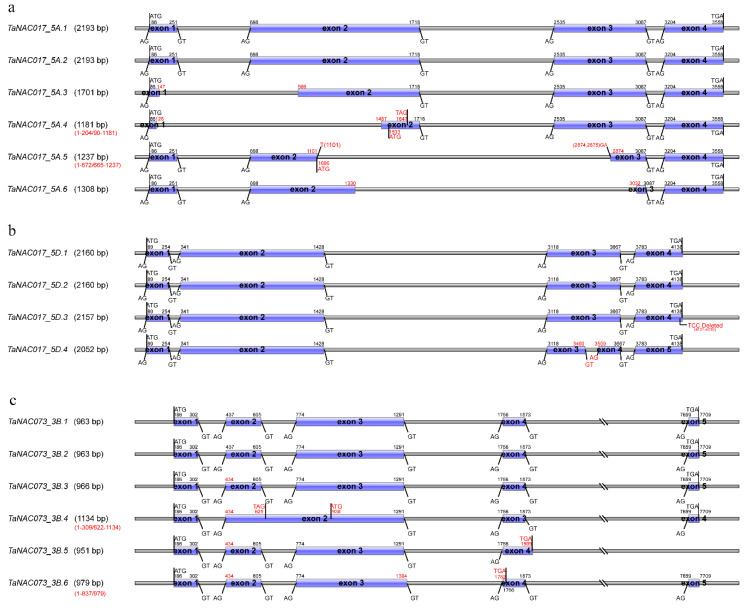
Structural patterns of 16 different transcripts from three TaNAC genes selected for description. (**a**) TaNAC017_5A corresponded to TraesCS5A02G271500, which was located on chr5A|481900414-481904347|. Open reading frames (ORFs) of TaNAC017_5A.1 and TaNAC017_5A.2 only had multiple single-nucleotide variations (SNVs). Four other transcripts were formed by deletion in the exon region. (**b**) TaNAC017_5D corresponded to TraesCS5D02G279100, which was located on chr5D|381032432-381036997|. TaNAC017_5D.2 could perfectly match TraesCS5D02G279100. TaNAC017_5D.1 had two SNVs in different loci with TaNAC017_5D.2. One SNV and a deletion of three bases were between TaNAC017_5D.3 and TaNAC017_5D.2. TaNAC017_5D.4 was TraesCS5D02G279100.2, which lacked an intron compared with TaNAC017_5D.2. (**c**) TaNAC073_3B corresponded to TraesCS3B02G410500, which was located on chr3B|647244571-647252470|.TaNAC073_3B.2 and TaNAC073_3B.3 had the same ORFs as TraesCS3B02G410500.2 and TraesCS3B02G410500.1, respectively; TaNAC073_3B.1 had one SNV with TaNAC073_3B.2; TaNAC073_3B.4 contained an intron, and ORF was divided into two segments; TaNAC073_3B.5 extended 36 bp bases of the following intron sequence after the fourth exon and lacked the last exon; TaNAC073_3B.6 continued to extend its third exon by 13 bp bases in the subsequent intron, which caused premature termination at Position 837 of the coding sequence. Notes: Exact sequence structures, including SNVs and corresponding to the structural pattern, are shown in Supplementary Figure S1. All details of these transcripts are given in Supplementary Table S3.

Analyzing these detected transcripts, we found sequence structural variations involving large fragments that could be classed into six types of alternative splicing: Alternative donor (A5/AD), alternative acceptor (A3/AA), A5 + A3, intron retention (IR), 3ʹ-terminal alternative splicing (TTS), and terminal exon skipping (TES). For example, TaNAC017_5A (Figure 3a) had six transcript variations due to A5 + A3 splicing. Compared to the longest transcript, TaNAC017_5A.1 or TaNAC017_5A.2, TaNAC017_5A.3 and TaNAC017_5A.6 had AS events happen in Introns 1 and 2, respectively. Interestingly, both of them encoded internally deleted peptides without shift coding at the N and C terminals. Notably, the encoding product of TaNAC017_5A.3 lost the NAM domain, but it was retained in TaNAC017_5A.6. Similar splicing events happened in TaNAC017_5A.4 and TaNAC017_5A.5. However, TaNAC017_5A.4 encoded an N-terminal-truncated protein due to a larger fragment splicing in Exons 1 and 2, while transcript TaNAC017_5A.5 obtained the property to splice TaNAC017_5A.1 into two truncated proteins, i.e., the N and/or C terminal of the full-length protein. Other examples of enhanced transcriptome plasticity were demonstrated by TaNAC017_5D, TaNAC073_3B, and TaNAC011_7D.1. TaNAC017_5D (Figure 3b), the homoeologous gene of TaNAC017_5A, produced an IR AS transcript in fungus-infected N9134, compared to the fully spliced transcript TaNAC017_5D.4 defined in IWGSC RefSeq v1.1. Intron retention commonly caused the shift-coding proteins. However, we found that IR transcripts could also normally encode a protein with 36 amino acids inserted in the C terminal. We also detected six transcript variants from the TaNAC073_3B gene (Figure 3c) that were caused by IR, A3, and TES AS. Of these, TaNAC073_3B.4 was an encoded N-terminal-truncated protein because the second intron was reserved. TaNAC073_3B.5 and TaNAC073_3B.6 also encoded variously C-terminal-truncated proteins due to TES and A5 + A3 events, respectively. In addition, in TaNAC011_7D.1 (Supplementary Table S3), the transcripts containing one more intron could be normally encoded, but the corresponding transcripts without the intron could not. Taken together, we could conclude that the wheat host activated the transcriptomic plasticity of NACs though the AS-enhancing ability to cope with fungal stress. Here, only limited examples of transcript variations were selected for inclusion in the main text, and the other sequence structural variations of the 186 transcripts examined in the present study are detailed in Supplementary Table S3.

To further understand the relationship between DE genes and transcription plasticity, we conducted comprehensive analysis (Table 2) of the 186 transcripts on the basis of DE and non-DE classification. We obtained interesting results on how they were produced, whether they could be encoded normally, and whether they responded to fungal stress. 

The total number of obtained transcripts was 117 and 69 from the selected 28 DE and 22 non-DE unigenes, respectively. The ratio according to the classification of DE/non-DE was 93:61 for normal-ORF transcripts (encoding full-length or longer proteins without shift coding), while the ratio of transcripts encoding truncated or shifted proteins was 24:8. Chi-squared test results (Table 2) demonstrate that the number of “abnormal” encoding transcripts from DE unigenes was significantly greater than that from non-DE unigenes (*p* = 0.030). In other words, the DE-TaNAC genes in wheat responding to fungi had a significantly higher proportion of “abnormal” coding transcripts (24/117 vs. 8/69, *p =* 0.0098; Supplementary Table S4) formed through alternative splicing etc. However, for normal encoding transcripts, there was no significant difference (Table 2 and Supplementary Table S4) between DE and non-DE genes.

### 3.5. DE-TaNAC Characterization Based on Phylogenetic Analysis, Conserved Domain, and Motif.s

**Figure 4:**
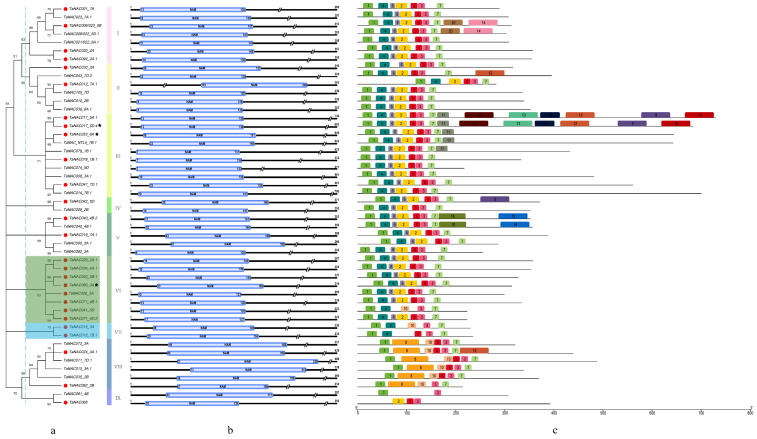
Phylogenetic classification, conserved domain pattern, and conserved motifs of 48 selected TaNAC transcription factors. (**a**) Maximum likelihood (ML) phylogenetic tree. Twenty-seven transcripts with red dots are from DEGs, others from non-DEG. Three transcripts with black pentagons were extracted from IWGSC RefSeq v1.1, and remaining 45 were cloned. (**b**) Domain patterns. For the sake of clarity, each sequence was truncated at different positions. (**c**) Motif patterns obtained using the Multiple Expectation Maximization for Motif Elicitation (MEME) suite. Sequence logos of Motifs 1–11 (except Motif 9) that had ≥5 sites shown in Supplementary Figure S2.

Transcripts belonging to the same gene or the same homoeologous gene cluster had few sequence–structure variations, and could be different in chromosome distribution. However, through preanalysis, we found that the relative evolutionary relationships of these transcripts were consistent, although there were a few differences. Therefore, based on the principle of selecting one transcript for each homoeologous gene cluster, we carried out phylogenetic analysis with 48 full-length coding TaNAC transcripts, which contained 27 DEGs and 21 non-DEGs under fungal stress. They were classified into nine subgroups of the maximum likelihood (ML) phylogenetic tree (Figure 4a). Figure 4b shows that the length of the conserved NAM domains of 48 TaNACs ranged from 110 to 170 aa, while the NAM domain usually (47 out of 48) existed in the N terminus, and the distribution spans were at Positions 6–226 aa. Furthermore, we detected seven conserved motifs from 48 NACs (Figure 4c and Supplementary Figure S2); among these, Motifs 1–6 were located in the NAM domain region. Motif 3 was irreplaceable in all tested TaNACs and the right boundary of the NAM domain. Combined with the phylogenetic tree, six transcripts in Subgroup VIII were characterized with the specific Motif 8, instead of Motifs 2, 4, and 6 (Figure 4). Motif 8 had some definite proprietary amino acid residues, such as HFFH and RKRRK (Supplementary Figure S2). Interestingly, all 10 TaNACs in Subgroups VI (including eight transcripts) and VII (including two transcripts) were classified as DEGs. Their NAM domains were usually followed by Motif 7, but the latter here was characterized by the conserved WV[L/V]CR amino acid residues (Figure 4c, Supplementary Figures S2 and S3). This hinted that these sequence structures may be implicated in specific responses against fungal stress in wheat.

### 3.6. Comparative Analysis of TaNAC Membrane-Associated Transcription Factors (MTFs)

Among the 559 TaNAC transcripts from IWGSC RefSeq v1.1, 36 NAC proteins harbored transmembrane motifs (Supplementary Table S1), and these were unevenly distributed among chromosomes. None was located in Chromosomes 1A, 1B, 1D, 4B, or 4D. These 36 transcripts were transcribed from 22 unique genes, of which 9 genes could transcribe 17 membrane-bound and 11 nonmembrane transcripts, while the 13 other genes only transcribed transmembrane-protein-encoding transcripts (19). Similarly, of the 154 normal encoding TaNAC transcripts from N9134, 19 had transmembrane motifs (Supplementary Table S3). Correspondingly, six of these transmembrane transcripts were transcribed from three unique genes, while the latter were also transcribed into corresponding AS nonmembrane transcripts. For example, TaNAC_NTL5_7B.1, TaNAC_NTL5_7B.2, and TaNAC_NTL5_7B.3 had transmembrane motifs, but TaNAC_NTL5_7B.4 did not. Similarly, TaNAC008_3A transcribed one transmembrane transcript (TaNAC008_3A.1) and one nonmembrane transcript (TaNAC008_3A.2) due to alternative splicing. This was similar for TaNAC047_7D.

From another perspective, we detected 12 membrane-bound transcripts from DEGs and 7 from non-DEGs in N9134 after fungi infection. There was no significant difference (Both *p*-values were much greater than 0.05.) in the ratio of membrane-bound transcripts when comparing classification of DEGs with non-DEGs under fungus stress (Table 3). However, the ratio of membrane-bound transcripts to nonmembrane transcripts from the fungus-infected N9134 was significantly greater than that from IWGSC RefSeq v1.1 (Table 4). To further dissect the functional properties, we observed the classification of all membrane-bound transcripts in the ML phylogenetic tree, and found that all seven transmembrane transcripts were clustered into Subgroup III. This hinted that an accompanying unknown motif or domain could be involved in membrane-bound TaNAC proteins, although this deduction needs further study. 

## 4. Discussion

The NAC family is one of the largest families of transcription factors, and it plays important roles in plant phenotypic morphogenesis by regulating downstream genes [28]. In general, these roles are so important and diverse that it is fundamental to understand them. The functional divergence of homologous NAC genes [37] suggests the necessity of separate studies on their functions and targets in different plants. Compared to plant development [38,39,40] and abiotic-stress responses [41,42], information on biotic-stress responses is limited, especially with regard to wheat response to stripe rust and powdery mildew. Here, we first focused on NAC gene transcription in wheat, and compared the difference between DEGs and non-DEGs. This offered a strong starting point from which to unravel the regulatory potential of NAC genes in response to fungal stress. Previous analyses showed that the NAC transcription regulatory domain (TRD) contains group-specific sequence motifs that are characterized by a high degree of intrinsic disorder [43]. Here, we found that WV[L/V]CR motif amino acid residues were conserved, immediately following the NAM domain. This gives a clue towards a more detailed understanding of the roles of specific NACs, but further experiments are needed. These resources could also be used to validate the potential specialization of NAC, especially in response to fungal stress in wheat.

### 4.1. Specific Spatiotemporal Expression and Alternative Splicing Suggested That TaNAC TF Family Transcripts Could Be Further Enriched and Improved

Gene pleiotropy is well acknowledged in classical genetics [44,45]. In the post-transcriptome era, we easily understand pleiotropy to be due to different transcripts generated via AS. In the present study, we cloned 167 full-length TaNAC transcripts from 77 gene loci. Among these, at least 79 transcripts were structural variations that were first detected in wheat plants with fungal infections. These extra transcripts enriched the coding structure of the wheat TaNAC genes. This means that the average number of transcripts in wheat under fungus stress was about 2.2 (167/77), which was significantly higher than 1.2 (559/460) in the reference genome (*p* = 2.01 × 10^−5^). Samples used in cloning were collected from 2.5 leaf-development stages under pathogen-infection conditions, but did not include different developmental samples and tissue types. It has been suggested that numerous NACs vary dramatically in different tissue types and developmental stages [38,46]. This hints that numerous TaNAC transcripts have not yet been identified, which warrants further exploration. The present study reported a variety of AS events in NAC TFs modulated during the interactions of wheat with *Bgt* and *Pst*. Different AS transcripts of the same TaNAC gene often contain changes at the 5’ terminals, 3’ terminals, and/or introns of sequences. This is bound to cause structural variations of coding proteins in the N- or C-terminal functional domains. It implies that TaNAC TFs responded to fungal stress not only through changes in the levels of expression [27,41,47,48], but also through transcriptional regulation to produce multifarious variants of transcripts with different coding sequences [43,49]. This substantiated our previous conclusion about AS in wheat [50], and suggested that the diversity of NAC transcripts should be considered in expression analyses and functional dissections. In addition, the TaNAC AS transcript number of DE genes was significantly higher than that of non-DE genes. Plants that produce AS isoforms are able to enhance their transcriptomic plasticity to cope with pathogens [50,51]. Considering the view that key TaNAC genes related to growth and development were relatively conserved in structure of transcripts [4,5,43], we inferred that TaNAC genes, with more AS events, may be related to stress responses. The expansion of NAC members may also be related to the self-regulation of function to adapt to environmental changes though orthologous exchange [52,53]. No matter whether these transcripts with structural variations were produced by fungal-stress regulation or carried by the materials, these NAC transcripts provide abundant information for a follow-up study on the relationship between structural variation and functional change. 

### 4.2. Small-Scale Duplications or Deletion Might Also Produce Structural Variations of Transcripts

In polyploid plants, both polyploidization events in evolution [52,53,54] and small-scale duplications [20,52,55] contribute to the expansion of multigene families, resulting in common wheat having more transcripts and more diversity of sequence variations than *Arabidopsis* or *O. sativa* do [19]. Here, eight TaNAC transcripts contained two normal NAM domains (Table 1), and each was a unique transcript of the gene locus in the second and third homologous groups. TraesCS5B02G271800 was the only gene in wheat to transcribe a transcript that had no NAM domain, and the NAM domain of TaNAC012_7A.1 presented at the C terminus (Figure 4b). It is not difficult to infer that these mutations are attributable to small-scale duplications or deletion, which produced some transcripts with special structures and then caused a loss or gain of function of transcription activities. The structurally variable transcripts of NAC in wheat were inconsistent with previous reports about the classical structural features of NAC TFs [4,5]. However, this supports the theory that loss or gain of function may occur in specific homeologs in TF families, and this is the same for sequence fragments [20,21]. In other words, these changes may enrich the functional plasticity of NAC TFs, although functional changes resulting from the positional changes, and the reasons for changes in sequence structure, need to be further studied.

### 4.3. Abnormalities in TaNAC Genes with Transmembrane Motifs Might Cause Different Functions by Varying Regulation

TFs were thought to be the regulators of gene expression, which means that they work intranuclearly [56]. However, some TFs were found to contain transmembrane motifs, which resulted in inactive regulating function, and they were called membrane-bound TFs [39]. Previous studies showed that NAC MTFs could be activated after transmembrane motifs were removed [39,57], and they could respond rapidly to sudden environmental changes because of the characteristics of membrane binding [57,58]. Here, we detected 36 membrane-bound transcripts (Supplementary Table S1), and divided them into two categories according to transcription from an AS or non-AS gene (producing a single transcript with transmembrane motif). In Category 1, NAC MTFs were accompanied with the corresponding transcripts without transmembrane motifs. This means that the activating transcripts coexisted with inactivating transcripts. The number of membrane-bound transcripts from N9134 was significantly greater than that from IWGSC RefSeq v1.1 (Table 4). Results from the current work suggested two possible reasons for this: (a) activating a NAC transcript directly modulates download genes that trigger a defense response in wheat and the inactivating NAC transcript is involved in other physical processes; (b) modulation of the defense pathway is conferred by balancing their expression levels. In Category 2, TaNAC genes only contained transcripts with transmembrane motifs, which meant that their activation was regulated by post-translational modifications after receiving a specific stimulus signal [59,60]. In addition, 2 of the 36 NAC MTFs, TraesCS2D02G378800.1 and TraesCS3A02G339600.2, had transmembrane motifs in the middle of the peptide instead of at the C terminus. This evidenced the diversity of sequence structures, and that the role of NAC is more complex than in model plants. Taken together, we concluded that wheat genomes have a complex regulating system, and that their detailed functions should be further studied, including dissecting NAC function in terms of pathogen-induced AS. Our results suggest that resistant wheat varieties undergoing fungal infection may trigger the corresponding defense mechanisms by regulating the AS of NACs, although functional characterization of these NAC genes is still in progress.

## 5. Conclusions

In the present study, through genome-wide reidentification of TaNAC genes in IWGSC RefSeq v1.1, a total of 101 high-confidence (HC) NAC transcripts were added and raised the number to 559 of TaNAC transcripts from 460 gene loci in wheat. And 180 new transcripts were obtained from the resistant hexaploid wheat line N9134 under fungal stress. Comparing with non-DE TaNAC genes, the DE-TaNAC genes in wheat responding to fungi had a significantly higher proportion of “abnormal” coding transcripts formed through alternative splicing etc. It suggested that *TaNACs* responded to fungal disease not only though regulating expression levels but also manipulating multifarious transcript variants. In addition, phylogenetic analysis discovered a potential amino acid residues WV[L/V]CR in Motif 7 following the NAM domain, which may be related to the specificity of TaNAC TFs responding to fungal stress. Furthermore, all the data for TaNAC responses to stripe rust and/or powdery mildew and their numerous structural variants provide a good resource for NAC function–mechanism analysis in the context of biotic-stress tolerance in wheat.

## Figures and Tables

**Table 1:** Conserved-domain classification of 559 high-quality Triticum aestivum NAC (TaNAC) transcripts identified from IWGSC RefSeq v1.1.

Number	Gene Names	Types
3	TraesCS5A02G271500.1, TraesCS5B02G271800.1, TraesCS5D02G279100.1	No domain
8	TraesCS2A02G326600.3, TraesCS2D02G101300.1, TraesCS2D02G336300.2, TraesCS4A02G213100.1, TraesCS5B02G480900.1, TraesCS5D02G481200.2, TraesCS6D02G266000.1, TraesCS7A02G152400.1	One short NAM domain
1	TraesCS1D02G004800.1	One normal NAM, one DnaJ, and one ZnF_BED domains
1	TraesCS7B02G461700.1	One normal NAM domain and two AA_Kinase domains
8	TraesCS2A02G328100.1, TraesCS2A02G363700.1, TraesCS2B02G381700.1, TraesCS2D02G334800.1, TraesCS2D02G361500.1, TraesCS3A02G269900.1, TraesCS3B02G303800.1, TraesCS3D02G269600.1	Two normal NAM domains
1	TraesCS2A02G326600.2	One normal NAM domain and one signal peptide in N-terminal
537	All other transcripts	One normal NAM Domain

**Table 2:** Conformity test between number of TaNAC transcripts and number of originally selected unigenes, including those classified as differentially expressed (DE) and non-DE under fungal stress.

No.	Types	DEG	Non-DEG	*p* Value
1	Selected unigenes	28	22	1.000
2	Total transcripts	117	69	0.058
3	Cloned transcripts	101	66	0.244
4	Assembly transcripts	9	2	0.085
5	Transcripts from assembly and IWGSC	7	1	0.073
6	Normal encoded transcripts	93	61	0.272
7	Cloned and normal encoded transcripts	78	59	0.826
8	Abnormal encoded transcripts	24	8	0.030 *
9	Cloned and abnormal encoded transcripts	23	7	0.023 *

Note: DEG: differentially expressed gene; p-values from chi-squared test calculated using ratio of DEG and non-DEG between each group and selected unigenes; * *p* < 0.05; ** *p* < 0.01.

**Table 3:** Conformity test between number of TaNAC transcripts with transmembrane motifs and number of originally selected unigenes according to classification as DE/non-DE under fungal stress.

Sequence Types	DEG	Non-DEG	*p*-Value
Selected unigenes from N9134	28	22	1.000
Transcripts with transmembrane motifs obtained from N9134	12	7	0.530
Transcripts with transmembrane motifs cloned from N9134	9	7	0.984

Note: *p*-values from chi-squared test calculated using ratio of DEGs to non-DEGs between each group and selected unigenes. * *p* *<* 0.05; ** *p <* 0.01.

**Table 4:** Conformity test between number of TaNAC transcripts with or without transmembrane motifs in N9134 and that in IWGSC RefSeq v1.1.

Transcript Types	With Transmembrane Motifs	Without Transmembrane Motifs	*p*-Value
Transcripts from IWGSC RefSeq v1.1	36	523	1.000
Total normal encoded transcripts from N9134	19	135	0.003 **
Cloned and normal encoded transcripts from N9134	16	121	0.012 *

Note: *p*-values from chi-squared test calculated using ratio of transcripts with/without transmembrane motifs between each group and that from IWGSC RefSeq v1.1. * *p* < 0.05; ** *p* < 0.01.

## Supplementary Materials

The following are available online at https://www.mdpi.com/2073-4425/11/9/1073/s1, Figure S1. Exact sequence structure corresponding to structural pattern in Figure 3. Figure S2. Sequence logos of Motifs 1–11 (except Motif 9) that had ≥5 sites. Figure S3. Conserved WV[L/V]CR amino acid residues of Motif 7 in sequences of Subgroups 6 and 7. Table S1. Various details of 559 reidentified high-quality TaNAC transcripts from IWGSC RefSeq v1.1. Table S2. Differential expression of 154 unigenes annotated as NAC candidates from RNA-seq data of N9134 infected with *Pst* and *Bgt*. Table S3. Various details of 186 TaNAC transcripts obtained from N9134 infected with *Pst* and *Bgt*. Table S4. Conformity test of ratio of normal and abnormal coding in TaNAC transcripts between two classifications of DEGs/non-DEGs under fungal stress. Table S5. Primers used for cloning TaNAC genes. Table S6. Primers used for qRT-PCR.
