# Supplementary figures and images for: Large-Scale Cloning and Comparative Analysis of TaNAC Genes in Response to Stripe Rust and Powdery Mildew in Wheat (Triticum aestivum L.)
Source: Genes (Basel). 2020 Sep 12;11(9):1073. doi: 10.3390/genes11091073 (PMC7564338; doi:10.3390/genes11091073)

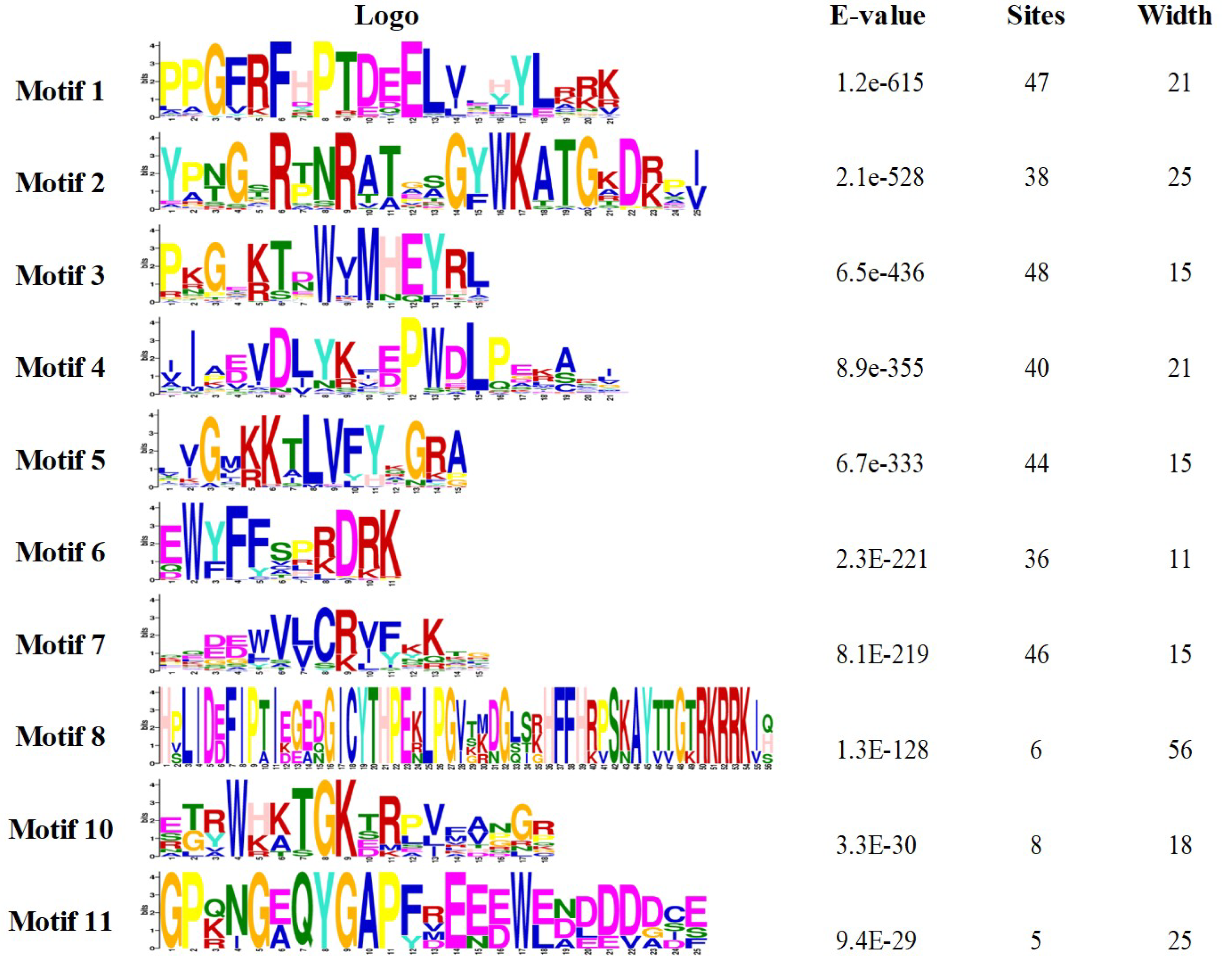

Supplement: Supplementary file 1 [file genes-11-01073-s001.zip › Figure S2. The sequence logos of motifs 1-11(but except motif 9) which had sites ≥ 5..tif]

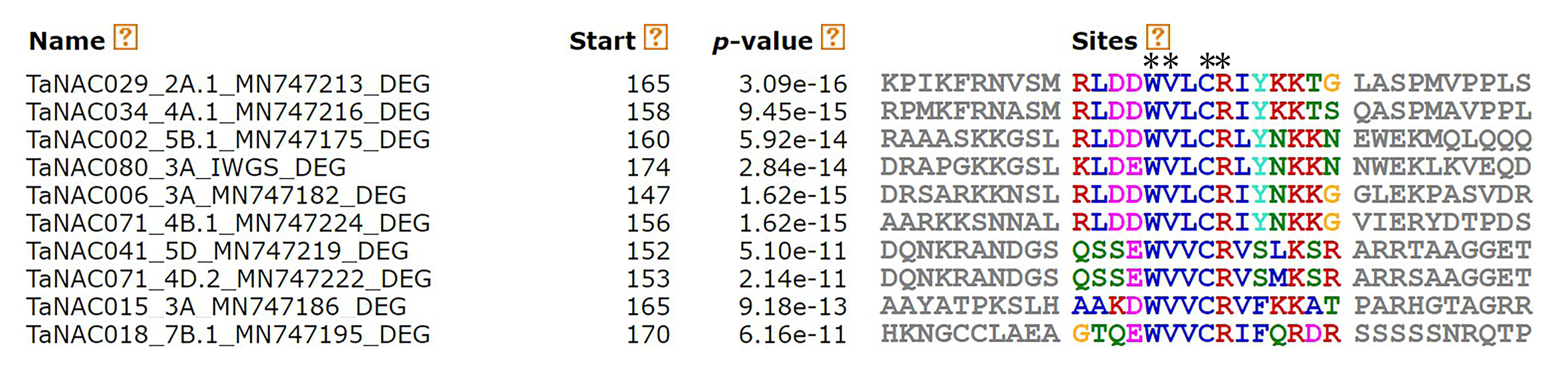

Supplement: Supplementary file 1 [file genes-11-01073-s001.zip › Figure S3. Conserved WV[L,V]CR amino acid residues of motif 7 in the sequences of subgroups 6 & 7..tif]
